# Does replacing sedentary behaviour with light or moderate to vigorous physical activity modulate inflammatory status in adults?

**DOI:** 10.1186/s12966-017-0594-8

**Published:** 2017-10-11

**Authors:** Catherine M. Phillips, Christina B. Dillon, Ivan J. Perry

**Affiliations:** 1 HRB Centre for Diet and Health Research, School of Public Health, Western Gateway Building, University College Cork, Cork, Ireland; 2 HRB Centre for Diet and Health Research, School of Public Health, Physiotherapy and Sports Science, Woodview House, University College Dublin, Belfield, Dublin 4 Ireland

**Keywords:** Physical activity, Inflammation, Obesity, Insulin resistance, Complement component C3, Moderate to vigorous physical activity, Pro-inflammatory cytokines, Adipocytokines

## Abstract

**Background:**

Sedentary behaviour, obesity and insulin resistance are associated with pro-inflammatory status. Limited data on whether physical activity modulates inflammatory status and counteracts obesity and insulin resistance associated low-grade inflammation exist. Our objective was to investigate associations between objectively measured physical activity and inflammatory status, and specifically whether substituting daily sedentary behaviour with light activity or moderate to vigorous physical activity (MVPA), is associated with beneficial alterations to the inflammatory profile among middle-aged adults and those at increased cardiometabolic risk (obese and insulin resistant subjects).

**Methods:**

Data are from a sub-sample of the Mitchelstown cohort; a population-based cross-sectional sample of 2047 Irish adults. Physical activity intensity and duration were measured in 396 participants for 7-consecutive days using the GENEActiv accelerometer. Isotemporal regression analysis examined the associations between replacing 30 min per day of sedentary behaviour with equal amounts of light activity and MVPA on inflammatory factors (serum acute-phase reactants, adipocytokines, pro-inflammatory cytokines and white blood cells (WBC)).

**Results:**

Reallocating 30 min of sedentary time with MVPA was associated with a more favourable inflammatory profile characterized by higher adiponectin and lower complement component C3 (C3), leptin, interleukin 6 (IL-6) and WBC concentrations (*P* < 0.05). No significant effects were noted with substitution of sedentary time with light activity. Among the obese subjects replacing sedentary behaviour with an equivalent amount of MVPA was associated with lower WBC counts (*P* < 0.05); no associations were detected among the insulin resistant (HOMA-IR >75th percentile) subjects. Among the non-obese and non-insulin resistant subjects substituting 30 min of sedentary behaviour with MVPA was associated with decreased C3, IL-6 and WBC concentrations.

**Conclusions:**

Replacing sedentary behaviour with MVPA modulates pro-inflammatory status. These findings, which highlight the need for the developing randomized trials aimed at lowering cardiometabolic risk, warrant further investigation.

## Background

Sedentary behaviour increases risk of obesity, type 2 diabetes (T2DM), cardiovascular disease (CVD), cancer, and shortens life expectancy [1–3]. Conversely physical activity exerts beneficial health effects including decreased risk of these conditions and reduced all-cause mortality [4–6]. Multi-factorial mechanisms, including the anti-inflammatory and insulin sensitising effects of physical activity and exercise, underlie the positive associations with cardiometabolic outcomes [7–10]. However, the supporting evidence is largely based on a limited range of inflammatory markers and self-reported physical activity. Objective and subjective methods, such as accelerometers and questionnaires, are currently the most commonly used methods to measure physical activity. Self-reported data are subject to recall and reporting biases, and poor agreement between objective and subjective measures of physical activity has been reported [11, 12]. Indeed the conflicting associations between self-reported and accelerometer based MVPA and CRP levels from the National Health and Nutrition Examination Survey (NHANES) underscore this issue [13, 14].

Insulin resistance and obesity are characterized by a low-grade but chronic inflammatory state [15]. We have recently shown that having a favourable inflammatory status [16] and a moderate to high level of physical activity [17] are both independently and positively associated with metabolic health status among obese middle-aged adults. Data on potential modulation of inflammatory status by physical activity in individuals with increased cardiometabolic risk are scarce. Data from the NHANES demonstrated an inverse association between objectively measured MVPA and CRP levels among diabetic adults [13]. The ATTICA study evaluated the relationship between self-reported leisure time physical activity and inflammatory markers in adults with metabolic syndrome (MetS) [18]. Relative to the sedentary MetS individuals those who were physically active had lower CRP, IL-6, tumour necrosis factor α (TNF-α) and WBC concentrations. Collectively these findings suggest that the benefits of physical activity, and particularly MVPA, on inflammatory status may be maintained, and perhaps even more evident, among individuals with metabolic perturbations.

Limited data on the relationship between objectively measured physical activity and inflammatory markers in adults, particularly those at increased cardiometabolic risk such as obese and insulin resistant individuals, are available. While the effects of replacing sedentary time with physical activity on cardiovascular risk biomarkers are now emerging [19], no data in relation to impact of substituting sedentary behaviour with different categories of activity on inflammatory markers exist. Therefore our primary aim was to determine the relationship between objectively measured physical activity intensity and duration with a range of inflammatory markers including acute-phase reactants, adipocytokines, pro-inflammatory cytokines and WBC counts measured at baseline in a cross-sectional sample of middle-aged adults, and specifically to investigate whether theoretical displacement of sedentary time with light activity or MVPA modulates inflammatory status among all subjects and those at increased cardiometabolic risk (i.e. obese and insulin resistant individuals).

## Methods

### Study design and subject recruitment

The Cork and Kerry Diabetes and Heart Disease Study (Phase II) was a single centre, cross-sectional study conducted between 2010 and 2011. A population representative random sample (Mitchelstown cohort) was recruited from a large primary care centre (Livinghealth Clinic) in Mitchelstown, County Cork, Ireland. Full details have been published elsewhere [20], but in brief Mitchelstown cohort participants were randomly selected from all registered attending patients in the 50–69 year age group. In total 3807 potential participants were selected from the practice list. Following exclusion of duplicates, deaths and ineligibles, 3043 were invited to participate in the study and of these 2047 (49.2% male) completed the questionnaire and physical examination components of the baseline assessment (response rate 67%). Of the 745 cohort participants invited to wear the accelerometer 475 subjects agreed to participate (response rate 64%). Ethics committee approval conforming to the Declaration of Helsinki was obtained from the Clinical Research Ethics Committee of University College Cork. All participants provided written informed consent.

### Accelerometer protocol

Objective physical activity levels were assessed using a GENEActiv accelerometer (ActivInsights Ltd., Cambridgeshire, United Kingdom). The technical reliability and validity of this accelerometer has been previously reported [21]. The tri-axial STMicroelectronics accelerometer with a dynamic range of +/−6 g (1 g = 9.81 m/s^2^) was attached to the participants’ preferred wrist with a strap [22]. Acceleration was sampled at 100 Hz and the accelerometer worn for 7-consecutive days. Following return of the accelerometer to the co-ordination centre data were extracted using GENEActiv software and then collapsed using sum of the vector magnitude (∑|√x^2^ + y^2^ + z^2^ - g|) into 60s epochs [21]. Each time interval, from the daytime wear-time (6 am-12 am) periods, was categorized as sedentary behaviour, light, moderate and vigorous activity and MVPA based on validated cut-off points for dominant and non-dominant wrist wear [23]. Of the 475 study participants 14 individuals had no data collected due to technical issues or non-return of the device and two were excluded based on location of wear i.e. leg. Following further exclusion of 63 participants with <10 h activity on any day, the remaining 396 subjects were included in the analysis.

### Clinical and anthropometric data

All participants attended the clinic in the morning after an overnight fast (minimum 8 h). Fasting blood samples were taken on arrival. Participants then completed a General Health Questionnaire and Food Frequency Questionnaire. Data on age, gender and lifestyle factors were gathered. Smoking status was defined as never, former and current smokers. Alcohol consumption included questions regards past and current intake to define moderate and non-moderate drinkers. Anthropometric measurements were recorded with calibrated instruments according to a standardized protocol. Body weight was measured in kilograms without shoes, to the nearest 100 g, using a Tanita WB100MA weighing scales (Tanita Corporation, IL, USA). Height was measured in centimetres to 1 decimal place using a Seca Leicester height gauge (Seca, Birmingham, UK). BMI was calculated. Waist circumference (defined as mid-way between lowest rib and iliac crest) was measured in centimetres to 1 decimal place using a Seca 200 measuring tape (Seca, Birmingham, UK). The average of two measures was used for analyses.

### Biological analyses

Plasma and serum were prepared from fasting blood samples. Fasting plasma glucose (FPG) was measured by Cork University Hospital Biochemistry Laboratory using fresh blood samples using a glucose hexokinase assay. Serum insulin, CRP, TNF-α, IL-6, adiponectin (ACDC), leptin, resistin and plasminogen activator inhibitor-1 (PAI-1) were determined using a biochip array system (Evidence Investigator; Randox Laboratories, Antrim, UK). C3 was determined by immunoturbidimetric assay (Rx Daytona; Randox Laboratories, Antrim, UK). WBC counts were determined by flow cytometry technology as part of a full blood count by the Cork University Hospital Haematology Laboratory. Homeostasis model assessment (HOMA), a measure of insulin resistance, was calculated as [(fasting plasma glucose x fasting serum insulin)/ 22.5] [24].

### Statistical analysis

Statistical analysis was conducted using PASW Statistics version 18.0 for Windows (SPSS Inc., Chicago, IL) and Stata (version 12, Stata Corp, College Station, Texas, USA). Individual median and 25th and 75th percentiles for average daily minutes spent in each activity category and inflammatory markers were calculated. ANOVA, t-tests and non-parametric tests were used to compare mean, % and median values, respectively, where appropriate. Two different linear regression models (single and isotemporal) were used. Single model analysis examined each activity intensity of sedentary behaviour, light activity and MVPA independently while isotemporal analyses examined each activity intensity while adjusting for time in other physical behaviours and total time. More specifically, the coefficient from an isotemperol model represents the estimated effects of substituting a specific activity intensity for the category dropped while holding total (wear) time and other activity constant [25]. Thus if we examine the effect of replacing sedentary behaviour with light activity we include light activity, MVPA and total wear time in the model whereas a model examining the effects of replacing MVPA with sedentary behaviour would include sedentary behaviour, light activity and total time. The isotemporal model is a linear regression model. While its primary function here is to determine whether substitution of different behaviours is beneficial to health it also examines whether this substitution relationship is linear. Thus the coefficients represent the effects for every 30-min increase substitution on each inflammatory marker. These models are described in greater detail elsewhere [26, 27]. Prior to entry into the models all intensity categories were divided by a constant of 30 such that a unit increase in the activity variable represented a 30 min increase in the given activity intensity. Three models were run for each inflammatory marker. The first model included age and gender. The second model additionally included smoking status, alcohol and energy intake. The third model additionally adjusted for BMI and anti-inflammatory medication use. An alpha level of 0.05 was set to evaluate significance.

## Results

### Inflammatory and physical activity profiles of the study sample and subgroups

A total of 396 male and female subjects were included in the analysis. Characteristics and inflammatory and physical activity profiles of the study sample and subgroups are presented in Table 1. The obese individuals had larger waist circumference, were more insulin resistant, comprised slightly less male subjects and had unfavourable inflammatory profiles characterized by higher C3, CRP, IL-6, leptin, resistin, PAI-1 and WBC, and lower ACDC concentrations (*P* < 0.05). Although there was a trend towards increasing TNF-α concentrations with increasing BMI this did not reach statistical significance. The insulin resistant individuals displayed greater BMI and waist circumference, comprised more males and in addition to all of the pro-inflammatory and pro-thrombotic alterations observed in the obese subjects, TNF-α concentrations were also elevated. No differences in age were noted between any groups. Average daily duration of sedentary behaviour, light activity and MVPA were significantly different within subgroups (*P* < 0.05). Obese and insulin-resistant individuals had higher sedentary behaviour and lower physical activity levels compared to non-obese and non-insulin resistant individuals.

**Table 1:** Characteristics and inflammatory and physical activity profiles of the study sample and subgroups

	Entire sample	Normal weight	Overweight	Obese	*P*	Non-insulin resistant	Insulin resistant	*P*
*n*	396	79	189	128		293	97	
Age (years)	59.58 ± 5.46	58.51 ± 5.43	59.73 ± 5.45	59.58 ± 5.43	0.13	59.49 ± 5.48	59.83 ± 5.44	0.61
Male (%)	46	64.6	54.5	46.9	0.046	41.7	56.7	0.01
BMI (kg/m^2^)	28.86 ± 4.55	23.25 ± 1.54	27.77 ± 1.35	33.95 ± 3.61	0.000	27.54 ± 3.61	32.85 ± 4.86	0.000
Waist (cm)	96.27 ± 13.39	82.80 ± 8.24	93.51 ± 0.00	108.70 ± 10.85	0.000	92.56 ± 12.04	107.29 ± 11.29	0.000
HOMA-IR	2.96 ± 3.25	1.49 ± 0.95	2.14 ± 1.50	5.11 ± 4.72	0.000	1.68 ± 0.80	6.87 ± 4.56	0.000
Inflammatory profiles								
C3 (mg/dL)	136.40 (120.30, 152.09)	115.68 (108.12, 138.27)	134.72 (121.63, 149.40)	147.60 (132.70, 166.90)	0.000	130.89 (115.87, 145.20)	152.52 (137.23,173.53)	0.000
CRP (ng/mL)	1.35 (0.99, 2.36)	1.02 (0.78, 1.55)	1.34 (1.03, 2.21)	1.68 (1.10, 3.06)	0.000	1.29 (0.97, 1.90)	1.95 (1.16, 3.34)	0.000
IL-6 (pg/mL)	1.78 (1.19, 2.97)	1.30 (0.90, 2.06))	1.67 (1.13, 2.62)	2.42 (1.63, 3.76)	0.000	1.53 (1.09, 2.53)	2.54 (1.69, 3.78)	0.000
TNF(pg/mL)	5.86 (4.73, 7.18)	5.67 (4.40, 7.10)	5.79 (4.67, 7.04)	6.05 (4.94, 7.82)	0.140	5.55 (4.59, 6.80)	6.89 (5.66, 8.65)	0.000
Leptin (ng/mL)	2.03 (1.25, 3.16)	1.38 (0.75, 2.00)	2.00 (1.14, 2.75)	3.01 (1.90, 4.87)	0.000	1.89 (1.14, 2.74)	2.78 (1.55,4.92)	0.001
ACDC (ng/mL)	4.86 (2.86, 7.54)	7.46 (3.89, 10.27)	5.03 (3.17, 7.65)	3.80 (2.45, 5.81)	0.000	5.48 (3.43, 8.24)	3.20 (2.25, 4.73)	0.000
Resistin (ng/mL)	4.95 (3.85, 6.54)	4.66 (3.68, 5.79)	4.85 (3.87, 6.55)	5.20 (3.99, 6.94)	0.019	4.79 (3.71, 6.31)	5.49 (4.39, 7.61)	0.007
PAI-1 (ng/mL)	25.53 (18.68, 33.31)	21.80 (16.13, 29.69)	24.27 (18.60, 33.34)	28.55 (19.98, 37.67)	0.005	23.24 (17.35, 31.17)	31.76 (24.49, 40.16)	0.000
WBC (10^9^/L)	5.60 (4.80, 6.70)	5.30 (4.40, 6.40)	5.50 (4.80, 6.50)	6.00 (5.30, 7.50)	0.011	4.60 (5.50, 6.50)	6.30 (5.40, 7.50)	0.000
Physical activity (mins per week)								
Sedentary	909 (853, 971)	894 (844, 970)	905 (843, 951)	945 (879, 991)	0.003	898 (840, 957)	952 (891, 994)	<0.0001
Light	103 (69, 135)	107 (68, 134)	108 (78, 138)	91 (58, 127)	0.04	109 (75, 141)	85 (55, 119)	0.0001
MVPA	62 (32, 103)	70 (42, 110)	67 (38, 104)	45 (23, 80)	0.0008	69 (38, 107)	45 (22, 68)	0.0001

Values are presented as mean ± S.D., %, and median (25th, 75th percentiles). ANOVA, t-tests and non-parametric tests were used to compare mean, % and median values, respectively, in subgroups according to BMI and HOMA-IR cut-offs. *ACDC* adiponectin, *C3* Complement component C3, *CRP* C reactive protein, *IL6* interleukin 6, *PAI-1* plasminogen activator inhibitor-1, *TNF-α* tumour necrosis factor α, *WBC* White blood cell

### Modulation of inflammatory status by physical activity intensity and duration

Table 2 presents the inflammatory profiles of the cohort according to tertiles of sedentary behaviour and physical activity intensity. Among all subjects increasing time spent in sedentary behaviour was associated with a more pro-inflammatory profile characterized by higher concentrations of C3, CRP, IL-6, TNF-α, leptin and WBC (*P* < 0.05). In contrast, lower C3, IL6 and leptin concentrations and lower WBC counts were noted with increasing levels of light activity (*P* < 0.05). Greater MVPA duration was associated with widespread favourable alterations to the inflammatory profile, most notably reduced levels of C3, CRP, IL-6, leptin and WBC (*P* < 0.05).

**Table 2:** Inflammatory profiles according to tertiles of sedentary behaviour and physical activity intensity among all subjects (*n* = 396)

	Sedentary			Light			MVPA		
	Tertile 1 *n = 132*	Tertile 2 *n = 132*	Tertile 3 *n = 132*	Tertile 1 *n = 132*	Tertile 2 *n = 132*	Tertile 3 *n = 132*	Tertile 1 *n = 132*	Tertile 2 *n = 132*	Tertile 3 *n = 132*
C3 (mg/dL)	126.0 (114.5, 143.6)	139.0 (127.2, 152.0)	143.5 (128.3, 162.8)*	139.7 (125.3, 156.9)	138.9 (124.4, 154.9)	129.6 (116.6, 147.2)*	143.5 (131.1, 163.9)	136.2 (118.7, 151.6)	128.3 (116.1, 144.4)*
CRP (ng/mL)	1.20 (0.91, 1.76)	1.57 (1.12, 2.26)	1.40 (1.02, 2.64)*	1.40 (0.98, 2.44)	1.30 (1.05, 1.98)	1.36 (0.96, 2.12)	1.47 (1.06, 2.88)	1.55 (1.09, 2.27)	1.17 (0.90, 1.70)*
IL-6 (pg/mL)	1.49 (1.04, 2.24)	1.67 (1.17, 2.81)	2.16 (1.44, 3.40)*	1.97 (1.34, 3.40)	1.61 (1.02, 2.54)	1.69 (1.25, 2.72)**	2.16 (1.37, 3.63)	1.67 (1.14, 2.91)	1.49 (1.04, 2.24)*
TNF-α (pg/mL)	5.49 (4.54, 6.9)	6.13 (5.13, 7.37)	6.01 (4.67, 7.22)**	6.04 (4.77, 7.22)	6.02 (4.86, 7.23)	5.59 (4.55, 7.07)	5.94 (4.82, 7.25)	6.13 (4.95, 7.22)	5.46 (4.55, 7.00)
Leptin (ng/mL)	1.63 (0.73, 2.42)	2.09 (1.34, 3.18)	2.43 (1.45, 3.88)*	2.17 (1.29, 3.54)	2.25 (1.37, 3.19)	1.74 (0.99, 2.66)**	2.44 (1.54, 4.31)	2.00 (1.27, 3.18)	1.72 (0.81, 2.42)*
ACDC (ng/mL)	4.92 (3.36, 7.79)	5.24 (2.97, 7.84)	4.65 (2.52, 6.77)	4.70 (2.40, 6.97)	4.90 (3.04, 7.46)	5.00 (3.23, 7.79)	5.18 (2.89, 7.13)	4.99 (3.71, 6.14)	4.89 (3.14, 7.60)
Resistin (ng/mL)	4.75 (3.91, 6.33)	5.06 (3.68, 6.63)	4.98 (4.05, 6.50)	4.88 (3.68, 6.46)	5.0 (3.89, 6.55)	4.97 (3.94, 6.59)	5.02 (4.12, 6.73)	4.99 (3.71, 6.41)	4.73 (3.76, 6.46)
PAI-1 (ng/mL)	23.8 (18.6, 32.5)	26.1 (19.5, 34.2)	26.1 (18.9, 33.3)	26.0 (17.5, 33.7)	26.3 (19.8, 33.6)	24.3 (17.7, 32.5)	25.8 (17.7, 33.5)	25.7 (19.6, 33.1)	24.9 (18.6, 33.3)
WBC (10^9^/L)	5.1 (4.4, 6.3)	5.6 (4.8, 6.4)	6.2 (5.4, 7.2)*	6.0 (5.3, 7.1)	5.6 (4.9, 6.5)	5.2 (4.6, 6.5)*	6.2 (5.4, 7.1)	5.6 (4.8, 6.4)	5.2 (4.4, 6.5)*

Values are presented as median (25th, 75th percentiles). *P* values were generated by comparing medians across groups using non-parametric tests. * represents *P* < 0.005 and ** represents *P* < 0.05. *ACDC* adiponectin, *C3* Complement component C3, *CRP* C reactive protein, *IL6* interleukin 6, *PAI-1* plasminogen activator inhibitor-1, *TNF-α* tumour necrosis factor α, *WBC* White blood cell

### Replacing sedentary behaviour with physical activity: Impact on inflammatory profile

Results from the isotemporal substitution modelling analyses are presented in Table 3. Reallocating time to MVPA from sedentary behaviour was linearly associated with beneficial modifications to the inflammatory profile. Adjusted for age and gender, more favourable C3, WBC, IL-6 and leptin concentrations were observed when 30 min of sedentary behaviour was substituted for 30 min of MVPA *(B = −3.59; 95% CI, −5.40, −1.78, B = −0.30; 95% CI, −0.43, −0.16, B = −0.32; 95% CI, −0.53, −0.11 and B = −0.24; 95% CI, −0.43, −0.05*) for C3, WBC, IL-6 and leptin concentrations, respectively. All of these associations remained significant in the further adjusted models, with the exception of leptin. No statistically significant associations were observed when 30 min of sedentary behaviour was substituted for light activity (*P* > 0.05).

**Table 3:** Association between sedentary behaviour and physical activity intensity with selected inflammatory markers among all subjects (n = 396)

	C3 (nmol/L)		WBC (10^9^/L)		IL-6 (pg/mL)		Leptin (mg/dL)		Adiponectin (ng/mL)	
Mins/day	β coefficients (95% CI)	Beta	β coefficients (95% CI)	Beta	β coefficients (95% CI)	Beta	β coefficients (95% CI)	Beta	β coefficients (95% CI)	Beta
Model A										
Sedentary	REPLACED				REPLACED		REPLACED		REPLACED	
Light	−0.25 (−2.25, 1.76)	−0.02	0.07 (−0.08, 0.22)	0.06	0.09 (−0.14, 0.32)	0.05	−0.04 (−0.25, 0.17)	−0.02	0.04 (−0.24, 0.33)	0.02
MVPA	−3.59 (−5.40, −1.78) ***	−0.26	−0.30 (−0.43, −0.16) ***	−0.29	−0.32 (−0.53, −0.11) **	−0.20	−0.24 (−0.43, −0.05) *	−0.16	0.24 (−0.03, 0.50)	0.11
Model B										
Sedentary	REPLACED		REPLACED		REPLACED		REPLACED		REPLACED	
Light	0.91 (−1.92, 3.74)	0.06	0.07 (−0.09, 0.23)	0.07	0.25 (−0.02, 0.51)	0.16	−0.12 (−0.41, 0.16)	−0.07	−0.28 (−0.66, 0.07)	−0.13
MVPA	−4.75 (−7.44, −2.07) **	−0.31	−0.28 (−0.43, −0.12) ***	−0.29	−0.41 (−0.67, −0.16) **	−0.28	−0.23 (−0.51, 0.04)	−0.14	0.43 (0.08, 0.77)*	0.19
Model C										
Sedentary	REPLACED		REPLACED		REPLACED		REPLACED		REPLACED	
Light	0.54 (−2.15, 3.23)	0.03	−0.06 (−0.10, 0.22)	0.06	0.23 (−0.04, 0.49)	0.14	−0.18 (−0.45, 0.09)	−0.10	−0.25 (−0.60, 0.10)	−0.11
MVPA	−3.27 (−5.89, −0.65) *	−0.21	−0.23 (−0.38, −0.07)**	−024	−0.34 (−0.60, −0.08)*	−0.23	−0.04 (−0.30, 0.22)	−0.04	0.25 (−0.09, 0.59)	0.12

Data are ß coefficients (95% CI) and beta values. *C3* Complement component C3, *IL6* interleukin 6, *WBC* White blood cell. Coefficients are based on average daily sedentary and physical activity duration. Results were expressed as regression coefficients that represented the change in outcome observed when 30 min of sedentary behaviour was substituted with 30 min of an alternative activity intensity (light activity or MVPA). Model A adjusted for age, gender and alternative physical behaviour intensities. Model B additionally adjusted for dietary intake, smoking status and alcohol consumption**.** Model C additionally adjusted for BMI and anti-inflammatory medication use. * represents *P* < 0.05 and ** represents *P* < 0.01, *** represents *P* < 0.0001

### Reallocating sedentary time with physical activity among high cardiometabolic risk groups

When stratified by BMI, replacing 30 min of sedentary behaviour with 30 min of MVPA was linearly associated with beneficial alterations to WBC counts among the obese *(B = −0.51; 95% CI, −0.91 to − 0.10*, *P* < 0.05) and non-obese subjects *(B = −0.21; 95% CI, −0.32, −0.08)* which remained significant in the fully adjusted model. Further positive associations with C3 *(B = −3.05; 95% CI, −5.09, −1.03)* and IL-6 *(B = −0.15; 95% CI, −0.30, −0.0007)* concentrations were observed among the non-obese subjects (Table 4). These associations remained significant in all models. When stratified by HOMA-IR, more favourable concentrations of C3 (*B = −2.43; 95% CI, −4.26 to − 0.60),* IL-6 *(B = −0.28; 95% CI, −0.42 to − 0.14*) and WBC counts (*B = −0.22; 95% CI, −0.39 to − 0.04*) were identified among the non-insulin resistant individuals following substitution of 30 min of sedentary behaviour for 30 min of MVPA (Table 5). These associations remained significant in all models. No significant findings were observed among the insulin resistant individuals (*P* > 0.05).

**Table 4:** Association between sedentary behaviour and physical activity intensity with selected inflammatory markers among non-obese subjects (*n* = 268)

	C3 (nmol/L)		WBC (10^9^/L)		IL-6 (pg/mL)	
	β coefficients (95% CI)	Beta	β coefficients (95% CI)	Beta	β coefficients (95% CI)	Beta
Model A						
Sedentary (mins/day)	REPLACED		REPLACED		REPLACED	
Light (mins/day)	0.54 (−1.87, 2.94)	0.03	−0.07 (−0.22, 0.08)	−0.07	0.12 (−0.05, 0.30)	0.11
MVPA (mins/day)	−3.05 (−5.09, −1.03) **	−0.24	−0.20 (−0.32, −0.08) **	−0.25	−0.15 (−0.30, −0.007) *	−0.16
Model B						
Sedentary (mins/day)	REPLACED		REPLACED		REPLACED	
Light (mins/day)	1.68 (−1.65, 5.02)	0.11	0.07 (−0.13, 0.27)	0.06	0.35 (0.09, 0.62) *	0.28
MVPA (mins/day)	−4.72 (−7.80, −1.63)**	−031	−0.24 (−0.43, −0.06) *	−0.26	−0.31 (−0.56, −0.06) *	−0.26
Model C						
Sedentary (mins/day)	REPLACED		REPLACED		REPLACED	
Light (mins/day)	1.67 (−1.69, 5.02)	0.10	0.07 (−0.13, 0.27)	0.07	0.34 (0.08, 0.61) *	0.26
MVPA (mins/day)	−4.69 (−7.83, −1.56) **	−0.32	−0.25 (−0.44, −0.06) *	−0.26	−0.30 (−0.55, −0.05) *	−0.25

Data are ß coefficients (95% CI) and beta values. *C3* Complement component C3, *IL6* interleukin 6, *WBC* White blood cell. Coefficients are based on average daily sedentary and physical activity duration. Results were expressed as regression coefficients that represented the change in outcome observed when 30 min of sedentary behaviour was substituted with 30 min of an alternative activity intensity (light activity or MVPA). Model A adjusted for age, gender and alternative physical behaviour intensities. Model B additionally adjusted for dietary intake, smoking status and alcohol consumption**.** Model C additionally adjusted for anti-inflammatory medication. * represents *P* < 0.05 and ** represents *P* < 0.01, *** represents *P* < 0.0001

**Table 5:** Association between sedentary behaviour and physical activity intensity with selected inflammatory markers among non-insulin resistant subjects (*n* = 293)

	C3 (nmol/L)		WBC (nmol/L)		IL-6 (pg/mL)	
	β coefficients (95% CI)	Beta	β coefficients (95% CI)	Beta	β coefficients (95% CI)	Beta
Model A						
Sedentary (mins/day)	REPLACED		REPLACED		REPLACED	
Light (mins/day)	0.98 (−1.13, 3.10)	0.07	0.15 (−0.01, 0.32)	0.14	0.18 (−0.02, 0.39)	0.13
MVPA (mins/day)	−2.43 (−4.26,-0.60) **	−0.20	−0.28 (−0.42, −0.14) ***	−0.29	−0.22 (−0.39, −0.04) *	−0.19
Model B						
Sedentary (mins/day)	REPLACED		REPLACED		REPLACED	
Light (mins/day)	1.98 (−0.95, 4.91)	0.13	0.15 (−0.02, 0.32)	0.15	0.31 (0.04, 0.58) *	0.23
MVPA (mins/day)	−3.48 (−6.22, −0.75)*	−0.26	−0.25 (−0.40, −0.09) **	−0.30	−0.32 (−0.57, −0.06) *	−0.25
Model C						
Sedentary (mins/day)	REPLACED		REPLACED		REPLACED	
Light (mins/day)	2.10 (−0.85, 5.05)	0.14	0.15 (−0.02, 0.32)	0.16	0.32 (0.04, 0.59) *	0.22
MVPA (mins/day)	−3.61 (−6.36, −0.85) *	−0.27	−0.25 (−0.41, −0.09) **	−0.30	−0.32 (−0.58, −0.06) *	−0.26

Data are ß coefficients (95% CI) and beta values. *C3* Complement component C3, *IL6* interleukin 6, *WBC* White blood cell. Coefficients are based on average daily sedentary and physical activity duration. Results were expressed as regression coefficients that represented the change in outcome observed when 30 min of sedentary behaviour was substituted with 30 min of an alternative activity intensity (light activity or MVPA). Model A adjusted for age, gender and alternative physical behaviour intensities. Model B additionally adjusted for dietary intake, smoking status and alcohol consumption**.** Model C additionally adjusted for BMI and anti-inflammatory medication. * represents *P* < 0.05 and ** represents *P* < 0.01, *** represents *P* < 0.0001

## Discussion

In this study, we demonstrated that increasing amounts of sedentary behaviour were associated with unfavourable alterations to the inflammatory profile among all subjects. Conversely increasing levels of light activity, and to a greater extent increasing MVPA, was associated with numerous beneficial modifications. Moreover, independent of potential confounders and time spent in other activities, replacing sedentary behaviour with MVPA among middle-aged adults was associated with a more favourable inflammatory profile characterized by lower C3, leptin, IL-6 and WBC concentrations.

Several possible mechanisms may explain the anti-inflammatory influence of physical activity, including reduction in visceral fat, enhanced insulin signalling and glucose transport with subsequent improved insulin sensitivity, increased expression of anti-inflammatory molecules and reduced toll-like receptor expression with subsequent decreased pro-inflammatory response [28–30]. The reported anti-inflammatory effects of physical activity have been largely based on self-reported physical activity and using CRP as a proxy for inflammatory status [2, 31–35]. Relatively little is known about the influence of both the intensity and duration of objectively measured physical activity on a range of inflammatory markers in middle-aged males and females, particularly among individuals at high risk of developing cardiometabolic disease. Recent examination of elderly men (mean age 78 years) from the British Regional Heart Study demonstrated more favourable IL-6 and CRP concentrations among those with higher levels of total physical activity, MVPA and light activity [36]. A novel finding in our study was that C3 concentrations were positively associated with increasing sedentary behaviour and negatively associated with increasing MVPA. Elevated circulating concentrations of C3, an acute-phase response protein with a central role in the innate immune system, have been associated with obesity and increased cardiometabolic risk [37]. We have reported novel genetic associations between *C3* polymorphisms with metabolic syndrome (MetS) risk [38] and that abdominal obesity and dietary fat modulate the relationship between elevated plasma C3 concentrations, *C3* genotype and MetS risk [39]. Although the relationship between C3 concentrations and cardiometabolic health has been examined, this has not been yet been investigated in the context of sedentary behaviour and physical activity in adults. The observed decreases in C3 levels with increasing MVPA (10%) and decreasing sedentary time (12%) are similar to the differences reported in incident ischemic event cases and non-cases [40], and in smokers with and without coronary heart disease (CHD) [41]. Similarly the differences observed in IL-6 levels with increasing light activity and MVPA (14 and 31% respectively) and decreasing sedentary time (31%) were greater than those reported between non-cases and cases of CVD and T2DM in the Caerphilly study [42], and between survivors of a first myocardial infarction and age and gender matched controls [43] for MVPA alone. Furthermore the differences in WBC levels comparing top and bottom tertiles of sedentary time, light activity, MVPA (16, 18 and 13% respectively) in the current work were greater than those reported between non-cases and cases of CVD [42]. Collectively these data suggest both physiologically and clinically significant changes in C3, IL-6 and WBC concentrations. To our knowledge the only examinations of C3 concentrations and objectively measured physical activity to date have been conducted in children and adolescents. Exercise, but not total physical activity, was related to C3 and CRP levels in 9 year old children [44], whereas total, vigorous and MVPA were not independently associated with C3 and CRP levels among adolescents [45]. In the HELENA study objectively, but not subjectively, measured vigorous physical activity was inversely associated with C3 levels in adolescents [46], highlighting the importance of more accurate objective measurement of habitual physical activity and well designed randomised clinical trials to assess the translation of such cross-sectional observational data to the clinical setting.

Isotemporal substitution modelling allows examination of the potential impact of reallocating a certain amount of time spent in sedentary behaviour with the equivalent amount of time spent in different physical activity categories, while keeping total time constant (e.g replacing 30 min sedentary time with 30 min MVPA). Although usually applied to cross-sectional data the causality caveat applies, however unlike standard regression models interpretation of the findings is more easily translatable in terms of the public health message and thus this methodology is becoming increasingly popular. Beneficial health effects of replacing prolonged sedentary time with MVPA have been reported. In an examination of 445 healthy men from the Whitehall II epidemiological cohort Hamer et al., determined the impact of replacing 10 min sedentary time (determined by waist worn accelerometer) with light activity and MVPA on HDL-C, tryglycerides, HbA1c and BMI [47]. Only MVPA was associated with favourable alterations for all markers. In a cross-sectional Swedish study of 836 middle-aged participants (52% women), progressively lower odds ratios for MetS risk were observed when replacing 10 min of sedentary time (determined by a hip worn accelerometer) with the same amount of light, moderate and vigorous activity (odds ratio (OR) 0.96 (95% confidence interval 0.93, 0.98, OR 0.89 (0.82, 0.97) and OR 0.41 (0.26, 0.66), respectively [48]. Buman et al.*,* recently reported beneficial associations with replacing 30 min/day sedentary time with light activity (improvements in triglyceride and insulin concentrations) and especially with MVPA (improvements to waist circumference, HDL-C, triglyceride, glucose and insulin concentrations and insulin sensitivity among a subsample (*n* = 923) from the cross-sectional 2005-2006 US NHANES [19]. Isotemporal substitution modelling has also recently been applied to longitudinal data. Using follow-up (mean 4.2 years) data from the 45 and Up study (*n* = 201, 129) Stamatakis et al., examined the impact of replacing 1 h of sedentary behaviour (sitting, television/computer screen time combined) with a range of activities (sleeping, standing, walking and MVPA) [49]. They reported lowest mortality risk with replacement of sedentary time with walking and MVPA. Consistent with these findings Schmid et al.*,* recently reported a 50% mortality risk reduction associated with reallocating 30 min of sedentary time with equal amount of MVPA [50]. Although some of these studies examined traditional CVD risk biomarkers, no study to date has investigated the impact of reallocating sedentary behaviour with physical activity on inflammatory markers. Importantly we show here for the first time that replacing 30 min of sedentary behaviour with MVPA is associated with significant reductions in C3 levels, as well as more favourable leptin, IL-6 and WBC concentrations in middle-aged adults. Collectively these data suggest that MVPA may be a potent health promoter. Indeed it is possible that such a favourable influence on inflammatory profiles may underlie some of the previously mentioned associations with cardiometabolic and mortality risk [48–50].

Insulin resistance and obesity are characterized by a low-grade but chronic inflammatory state, with elevated circulating levels of CRP, TNF-α, IL-6 and leptin and reduced ACDC concentrations associated with increased cardiometabolic risk [15, 51]. The pathway from obesity and insulin resistance towards overt T2DM represents a progressive phenotype, with raised inflammatory status frequently preceding T2DM by many years. Thus interventions to improve inflammatory status may attenuate progression from obesity and insulin resistance towards overt T2DM. Data from the NHANES demonstrated an inverse association between accelerometer derived objectively measured MVPA and CRP levels among diabetic adults [13]. In pre-diabetic adults increasing total and leisure time physical activity was associated with reduced IL-6 and increased ACDC concentrations [52]. Jennersjo et al., also demonstrated a negative association between IL-6 and CRP concentrations and daily number of steps recorded by pedometer in middle-aged adults with T2DM, with the greatest reductions observed among those achieving the recommended 10,000 steps a day [53]. In the current work objective measurement of physical activity over a 7-day period revealed a trend towards increased sedentary time and less time engaged in MVPA among the high cardiometabolic risk individuals. Although they displayed the most unfavourable inflammatory profiles, the isotemporal substitution analysis only identified a positive association between replacing sedentary behaviour with MVPA on WBC counts among the obese subjects, whereas substituting sedentary behaviour with MVPA was associated with reduced levels of C3, IL-6 and WBCs in both the non-obese and non-insulin resistant subjects. It may be that given the greater amount of time spent in sedentary behaviour among the high risk groups that a greater amount of sedentary time (> 30 min) needs to be displaced in order to see an impact on these inflammatory biomarkers among obese and insulin resistant individuals. These findings are consistent with the concept that sedentary behaviour is distinct from lack of MVPA (and vice versa) and has independent and different associations with metabolic health outcomes and thus should be treated as a separate entity [54]. Further research focussed on high cardiometabolic risk groups is warranted.

The main strengths of our study are the characterisation of the largest range of inflammatory markers examined in the context of sedentary behaviour, physical activity and isotemporal substitution analysis. Furthermore these biomarkers were determined at a commercial laboratory ensuring a high level of reproducibility. Secondly we used a valid and reliable accelerometer capable of objectively assessing time spent in sedentary, light, moderate and vigorous activity categories [21, 55]. In addition, this accelerometer collects data as raw acceleration and stores the data as g units for offline analysis. This allows efficient data cleaning, spurious data management, and application of various data processing algorithms post-data collection thereby increasing the transparency of the data and permitting more informed interpretation of the results. Notwithstanding these strengths some limitations can be identified. The cross-sectional study design limits inference regards causality and although we controlled for confounding factors we cannot exclude the possibility that unmeasured confounders such as genotype may also influence our observations. Physical activity data were collected over a one-week period which may not reflect habitual physical activity levels over a longer time period. Compared with self-report, objective measurement of physical activity using accelerometers is more precise and less biased (particularly with respect to recall and social desirability bias) with reduced potential for measurement error and misclassification bias. However potential sources of bias such as reactivity and social desirability, which may cause the accelerometer wearer to change their usual physical activity level, may still remain. The stratification of analysis by subgroups resulted in small sample sizes, thus sub-group analyses may not have had sufficient power to determine true associations between physical behaviour and inflammatory status. Generalizability of our findings may also be limited. The Mitchelstown cohort (response rate 67%) was a random sample of middle-aged adults from an area representative of both urban and rural population in Ireland. Our previous research suggests that approximately 98% of Irish adults are registered with a GP and that, even in the absence of a universal patient registration system, it is possible to perform population based epidemiological studies that are representative of the general population using these methods [56]. Within the sub-sample examined in the current analysis women were more likely to agree to wear the accelerometer. However it should be noted that there were no statistically significant differences in age, gender or BMI between all subjects included (*n* = 398) and excluded (*n* = 77) in the final analysis.

## Conclusions

Among middle-aged adults sampled from primary care, increasing amounts of objectively measured physical activity (light activity and particularly MVPA) showed favourable associations and sedentary behaviour detrimental associations with a range of inflammatory biomarkers. Furthermore our results provide evidence that replacing sedentary behaviour with MVPA is associated with beneficial modifications to the inflammatory profile, suggesting that MVPA may be an important potential modifier of adverse inflammatory profiles. These findings represent an important contribution to the knowledge base and warrant further investigation. They provide justification for the design of randomized trials aimed at replacing a portion of sedentary behaviour with physical activity which will help generate evidence to guide policy in this field.

## Abbreviations

ACDC: Adiponectin
BMI: Body mass index
C3: Complement component C3
CVD: Cardiovascular disease
FPG: Fasting plasma glucose
GHQ: General health questionnaire
HDL-C: High density lipoprotein cholesterol
HOMA-IR: Homeostasis model assessment of insulin resistance
IDL: Intermediate-density lipoprotein
LDL-C: Low density lipoprotein cholesterol
MetS: Metabolic syndrome
MVPA: Moderate to vigorous physical activity
PAI-1: Plasminogen activator inhibitor-1
T2DM: Type 2 diabetes mellitus
TG: Triglyceride
Total-C: Total cholesterol
WBC: White blood cell
